# Analysis of land subsidence caused by hydrodynamic force in Loess Hilly and gully region based on SBAS-InSAR

**DOI:** 10.1371/journal.pone.0279832

**Published:** 2023-01-26

**Authors:** Xuan Liu, Chao Ma, Han Ling, Weitao Yan, Hebing Zhang, Xuhai Jiang

**Affiliations:** 1 School of Surveying and Land Information Engineering, Henan Polytechnic University, Henan Jiaozuo, China; 2 School of Resources and Environment, Henan Polytechnic University, Henan Jiaozuo, China; 3 Shaanxi Key Laboratory of Land Consolidation, Shaanxi, Xi’an, China; 4 College of Land Engineering, Chang’an University, Shaanxi, Xi’an, China; Jinan University, CHINA

## Abstract

After large-scale land consolidation in hilly loess region of the Loess Plateau in China, land subsidence has a wide affecting area and considerable difficulty of prevention. Hence, large-scale, stabilized, and continuous deformation monitoring is urgently needed for slopes. In this study, land consolidation zone in the loess platform area of Weinan, China, was selected as the object, and the 30-scene Sentinel-1A data in Jan, 2018 to Dec, 2019 were analyzed. The mean annual velocity of ground deformation was from -6.19 mm∙a^-1^ to 3.86 mm∙a^-1^, and Accumulated deformation velocity was within -8.49 mm∙a^-1^ to 7.24 mm∙a^-1^. Accumulated deformation of land consolidation changed with the seasons changing. The interrelationship between the spatiotemporal variations in ground subsidence and the precipitation, ground water, loess engineering properties was also discussed. Accumulated deformation of land consolidation changed with the seasons changing. The precipitation accelerated the subsidence by unexpected strong precipitation reflects that the infiltration of rainwater can lead to compacted loess deformation which caused by moistening effect. Under varying ground water environment, external loads may lead to soil collapse, resulting in non-uniform land subsidence. Co-compression deformation of original loess and compacted loess is main influencing factors of subsidence. These findings have important implications and significant positive effects on the prevention of potential hazard such as subsidence and side slope slip.

## Introduction

The Loess distribution in the Loess Plateau in China is highly representative and the loess is characterized by fine soil particles, homogeneous texture, and loose structure. Under the combined effects of wind, water and gravity, soil erosion has been continuously exacerbated. Meanwhile, human activities have significant effects on soil erosion. As a result, unique hilly loess regions are observed in the Loess Plateau [1]. The hilly loess regions are characterized by vertical and horizontal gullies and complex and uneven terrain. Therefore, soil erosion and vegetation damage in these regions are severe. Indeed, the hilly loess regions are one of the areas with the most severe soil erosion and induced damages globally [2]. In order to increase the area of cultivated land, guarantee food security, restore the ecological environment and increase farmers’ income, overall improvement of gully land in hilly loess region characterized by gully termination and land consolidation, which is denoted as the “gully control and land consolidation” project, has been applied in various regions in the Loess Plateau [3]. The “gully control and land consolidation” project mainly involves construction of farmland in hilly loess region of the Loess Plateau gullies, as well as other structures such as sediment storage dams, flood discharge channel, steep slope, and scarp. This project aims to ensure slope stability for post-treatment gully land, prevent natural disasters such as soil erosion, and guarantee sustainable utilization of land resources [4].

Nevertheless, land use activities in the Loess Plateau have been rapidly increasing. On the one hand, these activities lead to increased probability of geological disasters such as landslide, collapse and debris flow. On the other hand, manual excavation may change the original shape and internal stress field of loess slope. Meanwhile, inappropriate mechanical slope cutting tend to cause land subsidence, resulting in slope deformation [5], vegetation damage [6], surface collapse [7], and building deformation [8]. For this reason, effective land utilization is barely possible in areas exposed to gully control and land consolidation. Hence, after large-scale gully control and land consolidation in hilly loess region of the Loess Plateau, land subsidence has a wide affecting area and severe difficulty of prevention. Also, land subsidence in this case is challenging to be recovered. In order to prevent continuous effects on areas exposed to gully control and land consolidation, stabilized and continuous deformation monitoring is urgently needed for slopes.

The interferometry synthetic aperture radar (InSAR) method is an effective way to monitor surface deformation [9]. It can obtain real time, high-resolution terrain and surface deformation in an all-weather, large area and non-contact way and achieve high-precision detection of minor surface deformation [10]. To date, InSAR plays a key role in identification and investigation of geological disasters and monitoring of slope stability [11, 12]. D-InSAR is a satellite-based remote sensing technique that can be used to measure surface displacement on the Earth. Applying D-InSAR, phase measurements from radar images acquired over the same region at different times are used to precisely measure relative distances [13]. D-InSAR is sensitive to very small changes and can measure displacement with accuracies ranging from millimeters to centimeters under ideal conditions. Compared with the D-InSAR method, the small baseline subset (SBAS-InSAR) method is free from the effects of temporal, spatial baseline decoherence and atmospheric effects [14]. The measurement capability and high precision of the SBAS method are convenient for further exploring the correlation between different factors and deformation phenomena to learn about the internal mechanism of the deformation process [15]. The SBAS method, as a representative time-series InSAR technique, has derived a variety of improved versions to solve different problems. Because of its excellent ability to survey ground displacement, this technique is suitable for different application scenarios such as monitoring and warning of landslide [16] land subsidence [17] and deformation [18].

In this study, the SBAS-InSAR method was employed for subsidence monitoring in hilly loess regions of the Loess Plateau. Here in the land consolidation zone in the loess platform area of Weinan, China, was selected as the target area and the Sentinel-1A data of the 30-scene C band from Jan, 2018 to Dec, 2019 were used. The land subsidence in the target area was investigated using the SBAS-InSAR method. Additionally, the reasons behind land subsidence in the target area were thoroughly investigated based on geological, hydrological, and meteorological data.

## Method

### SBAS-InSAR method

Using SAR datasets, the SBAS-InSAR method satisfies interference image pairs corresponding to thresholds of time and space baselines. Herein, residual orbit errors and other noises were eliminated by unwrapping, filtering, and offset estimation, followed by removal of residual terrain phases. Equations were established based on reliable highly coherent points [19] and solved by the singular value decomposition (SVD) method to estimate the deformation rate on each highly coherent point. By spatial and temporal filtering, atmosphere and nonlinear deformation phases were separated from residual phases and deformation series covering the entire observation period were obtained [20]. The procedures are as follows:

Assuming that N+1-scene SAR images (*t*_1_, *t*_2_, ⋯ *t*_*n*−1_, *t*_*n*_) are present, one of them was selected as the master image, while registration and re-sampling were applied for other N-scene auxiliary images. After setting of thresholds of time and space baselines, baseline combination was achieved according to the small baseline principle to obtain M interference image pairs satisfying thresholds of time and space baselines. Herein, M satisfies:

(N+1)/2<M<N(N+1)/2 (1)

For any of the M interference image pairs, tA and tB (tA > tB, reference time = t1) are defined as the time for acquisition of main and auxiliary images, respectively. Each interference image pair was interfered, flattened, de-terrained, filtered and unwrapped. In this way, the phase value of corresponding highly coherent point (*x*, *r*) can be expressed as:

$$\delta \phi_{j}=\phi_{B}(x,r)-\phi_{A}(x,r)\approx \frac{4\pi }{\lambda }[d(t_{B},x,r)]-[d(t_{A},x,r)]+\Delta \varphi_{i}^{topo}(x,r)+\Delta \varphi_{i}^{res}(x,r)$$ (2)

where *j* ∈ (1, ⋯, *m*), *ϕ* refers to the interference phase, *λ* refers to the wavelengths, *d* refers to the deformation along the line of sight, which can be expressed as:

$$d(t_{B},x,r)-d(t_{A},x,r)=v_{i}(t_{B}-t_{A})$$ (3)

It can be changed to a matrix:

Bv=δϕ (4)

where B is a matrix with dimensions of M×N. If a single interferogram subset is formed, the least-squares method can be used to solve the deformation rate. When two or more interferogram subsets are created, the singular value decomposition (SVD) method with minimum norm conditions needs to be introduced to obtain the deformation rate. Of course, in this case the SVD decomposition is applied to the matrix B, and the minimum-norm constraint for the velocity vector *v* does not imply the presence of large discontinuities in the final solution; obviously, an additional integration step is necessary to achieve the final solution *ϕ*.

### Interferometric coherence analysis

SBAS-InSAR ground deformation detecting is implemented by converting the phase difference into deformation, based on the phase difference measurement between radar waves. Phase difference aims to acquire the spatial geometry information, but the phase signal collected by the radar image contains the actual path-related information and plenty of noise signals. Therefore, the SC coefficient is a crucial parameter to evaluate the quality of the interferogram between different SAR image pairs[21]. The model of the spatial coherence coefficient can be expressed by:

$$\gamma_{spt}=\frac{\sum_{n=1}^{N}\sum_{m=1}^{M}u_{1}(n,m)u_{2}^{*}(n,m)}{\sqrt{\sum_{n=1}^{N}\sum_{m=1}^{M}|u_{1}(n,m){|}^{2}\left|u_{2}(n,m)\right|}}$$ (5)

where * represents complex conjugate, *u*_1_(*n*, *m*) and *u*_2_(*n*, *m*) represent the complex values at (*n*, *m*) which is the image coordinate in the master and slave image data blocks, |*u*_1_(*n*, *m*)|^2^ and |*u*_2_(*n*, *m*)|^2^ represent the second-order norm of the data, *M* and *N* represent the sizes of the data block for calculating the coherence, *n* and *m* are the row and column numbers in the data block, then it takes any pixel as the center and calculates the coherence coefficient according to the window size of *n* × *m*.

## Background of target area and data source

### Background of target area

**Fig 1:**
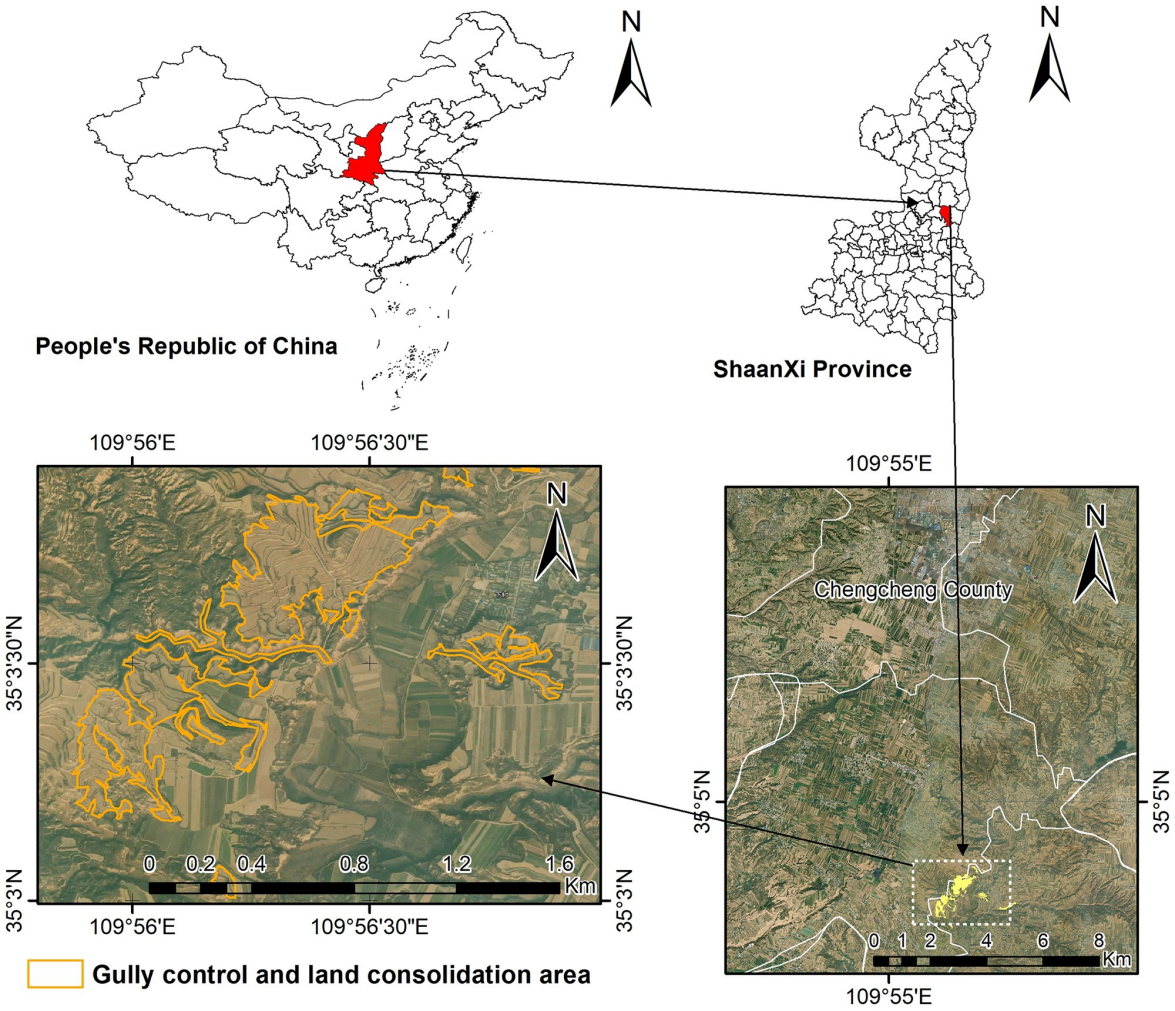
Geographical location of the target area. Source: USGS Earth Resources Observatory and Science Center (https://www.usgs.gov/centers/eros).

The target area, which has an overall area of 45.16 hm^2^, is located in the southeast part of Chengcheng County, Weinan, China. Its geographical coordinates are E109°55′49″-E109°57′27″ and N35°3′7″-N35°3′58″. The climate of the target area is semi-humid monsoon climate in warm temperate zone with annual average temperature of 12°C, annual precipitation of 680 mm, frost free period of 204 d and annual sunshine hours of 2600 h. Additionally, the target area has large temperature difference between day and night. The entire target area is in hilly loess region of the Loess Plateau. Located to the south of the Huanglong Mountain, the target area has altitudes decreasing from north to south (maximum and minimum altitudes are 1265.7 m and 470 m, respectively). It is characterized by vertical and horizontal gullies and high tablelands. Indeed, the target area has 98 gullies and the maximum altitude difference between gullies is 194 m. The county is divided into three ‘Liang’ (narrow hillock) and one ‘Yuan’ (tableland) by four main gullies and rivers flowing through it include Dayu River, Xianxi River, Changning River, Kongzuo River, and Macun river, all which merge into Luohe River at the southwest part. The main landforms of the target area include low mountains in the north part, alluvial fans in front of mountains, gullies, loess narrow hillocks, and loess tablelands. (See Fig 1).

### Datasets and processing

According to imaging conditions of the space-based radar covering the target area, a total of 30 Sentinel-1A IW SLC images provided by European Space Agency (ESA) with the resolution of 5 m×20 m (range×azimuth) is selected, and its time-span from Jan, 2018 to Dec, 2019. The Digital Elevation Model (DEM) data of the study area is generated through the Shuttle Radar Topography Mission (SRTM) with a resolution of 30 m provided by National Aeronautics and Space Administration (NASA). Besides, the Precise Orbit Data of Sentinel-1A is provided by ESA. And GACOS data is used to correct the atmospheric delay errors. The detail information of the images is shown in Table 1.

**Table 1:** The SAR datasets information.

Satellite	Time-span	Volume of images	Azimuth angle (°)	Off-nadir angle (°)	Resolution	Number of interferograms
Sentinel-1A	20180130–20191215	30	-10.4 (Ascending)	33.7	5 m×20 m	435

**Fig 2:**
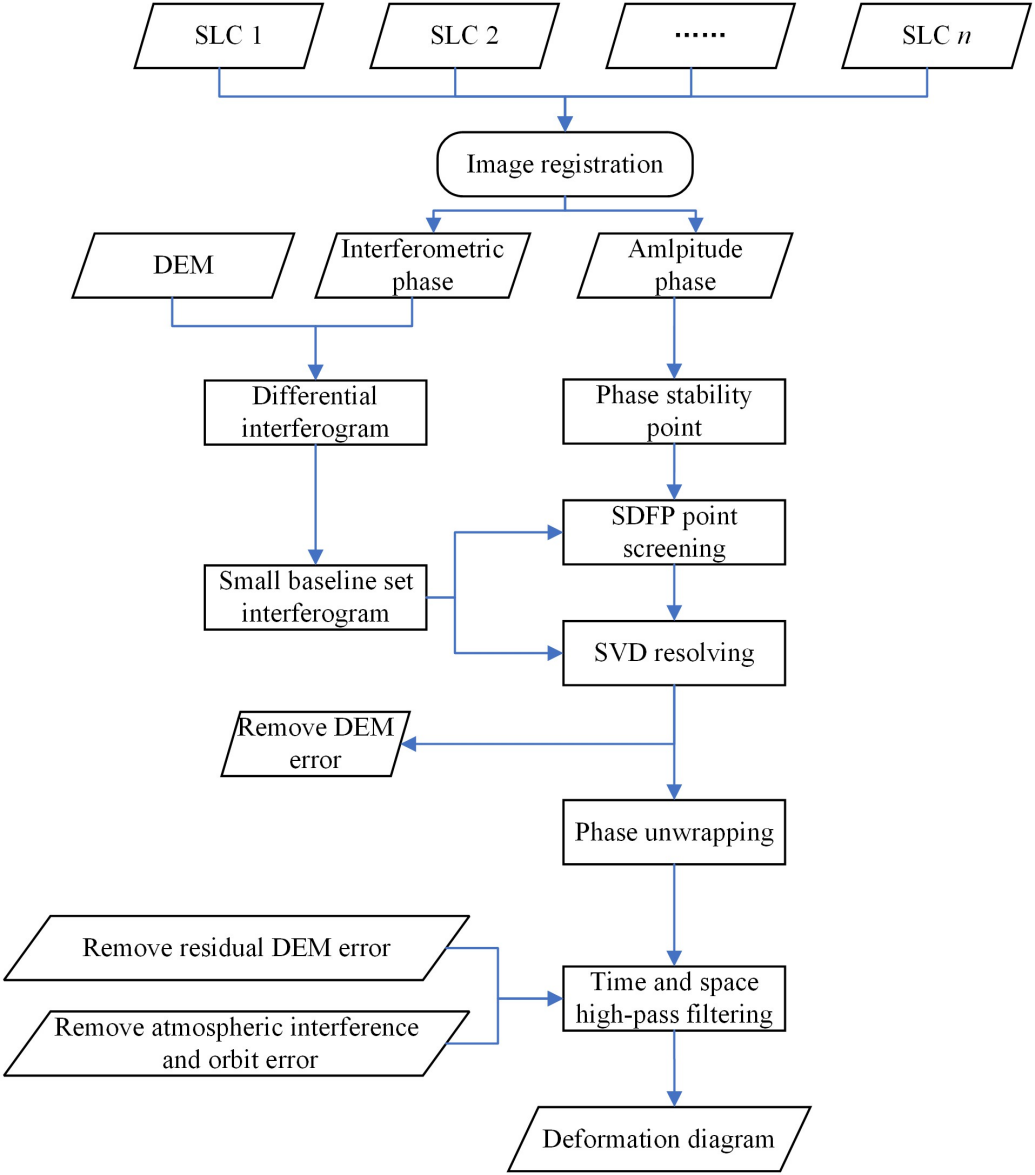
Interference phase combination.

The data were processed using the SBAS time-series analysis model, as shown in Fig 2 [22].

Original loess and compacted loess in the gully control and land consolidation region were taken samples including original loess 1 sample and compacted loess 3 samples. The physical properties at the different location’s samples were test in laboratory. The ground water tables of two wells in the target area were monitored and time-series surface deformations at these locations were extracted to analyze the temporal correlation of ground water table and land subsidence.

The time series deformation monitoring values from 2018 to 2019 in 8 GPS stations were selected for accuracy evaluation. In order to unify the InSAR processing results and GPS observations in the research period, the image data from 2018-02-23 to 2019-12-15 was used to generate the average velocity graph in the sight direction obtained by InSAR. The InSAR processing results corresponding to each GPS point are extracted and compared with the GPS deformation in the sight direction obtained by InSAR. A stable reference point is set during the processing.

**Fig 3:**
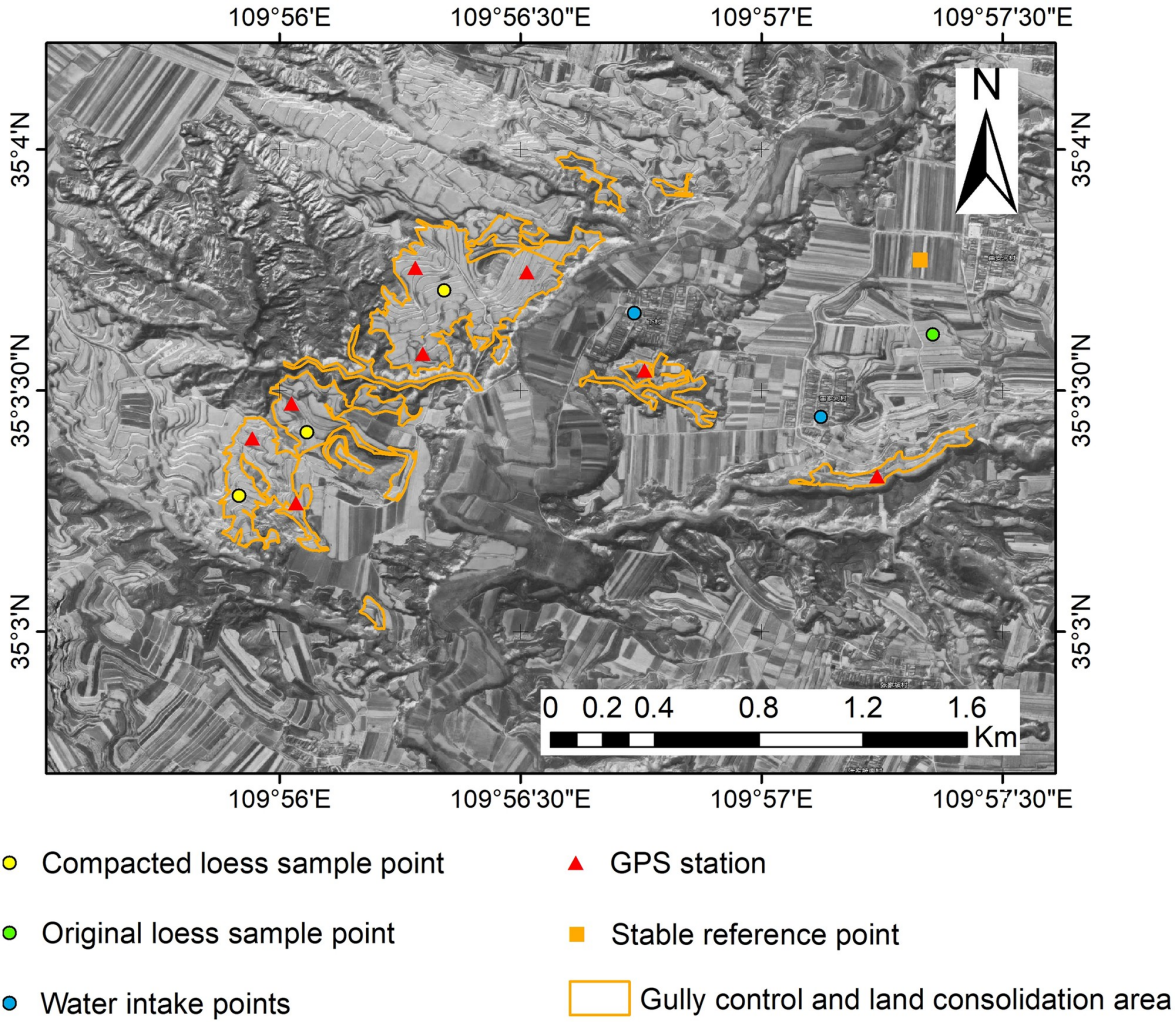
Spatial distribution of different data sampling points.

The water intake points, original loess and compacted loess sample points, stable reference point and GPS station are shown in Fig 3.

By considering critical values of time vertical baseline, space vertical baseline and Doppler centroid frequency baseline, as well as effects of the two factors with minimum sum of absolute values, the image obtained on Jan 30, 2018 was set as the master image and the thresholds of time and space baselines were set to be 650 d and 200 m, respectively, for time and space baseline combination. In this study, the maximum time and vertical baselines were 684 d and 39 m, respectively. All of the SAR image phase unwrapping by Delaunay method. 20 to 30 points with good coherence for GCP Selection were selected in the Interferogram after unwrapping for refinement and reflattening.

**Fig 4:**
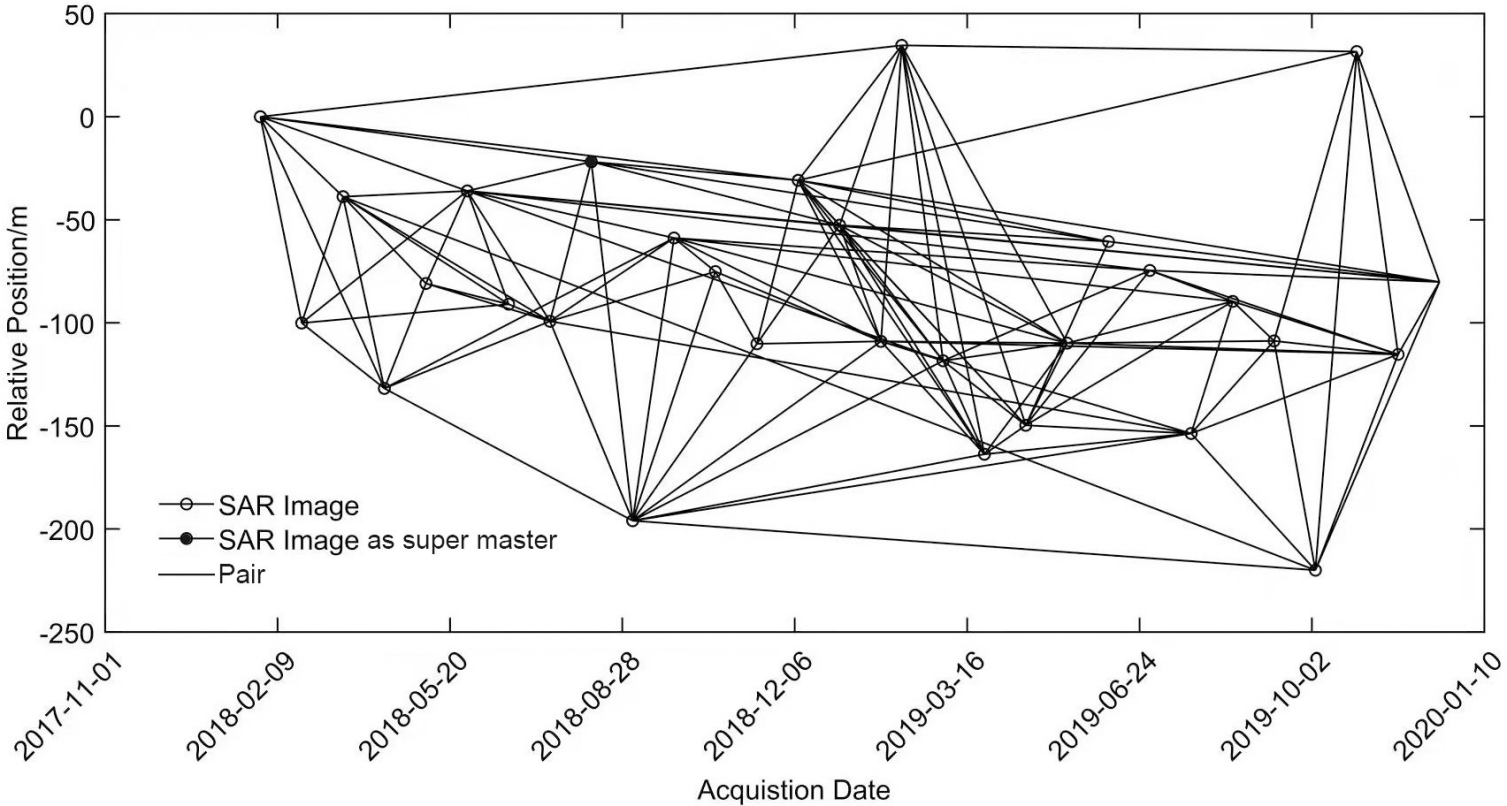
Procedures of time-series analysis of SBAS-InSAR.

Fig 4 depicts the distribution of time and space baselines. The terrain phases were removed using SRTM DEM (resolution 30 m), followed by differential interfering, deflattening, filtering and unwrapping. In this way, 135 interference pairs were eventually obtained. Geocoding was achieved using orbit data and DEM-processed data to obtain the differential interference phase map. To minimize effects of other factors such as noises, the coherent mass of each interference pair was examined and interference pairs with poor coherence were removed before extraction of target coherent points.

**Fig 5:**
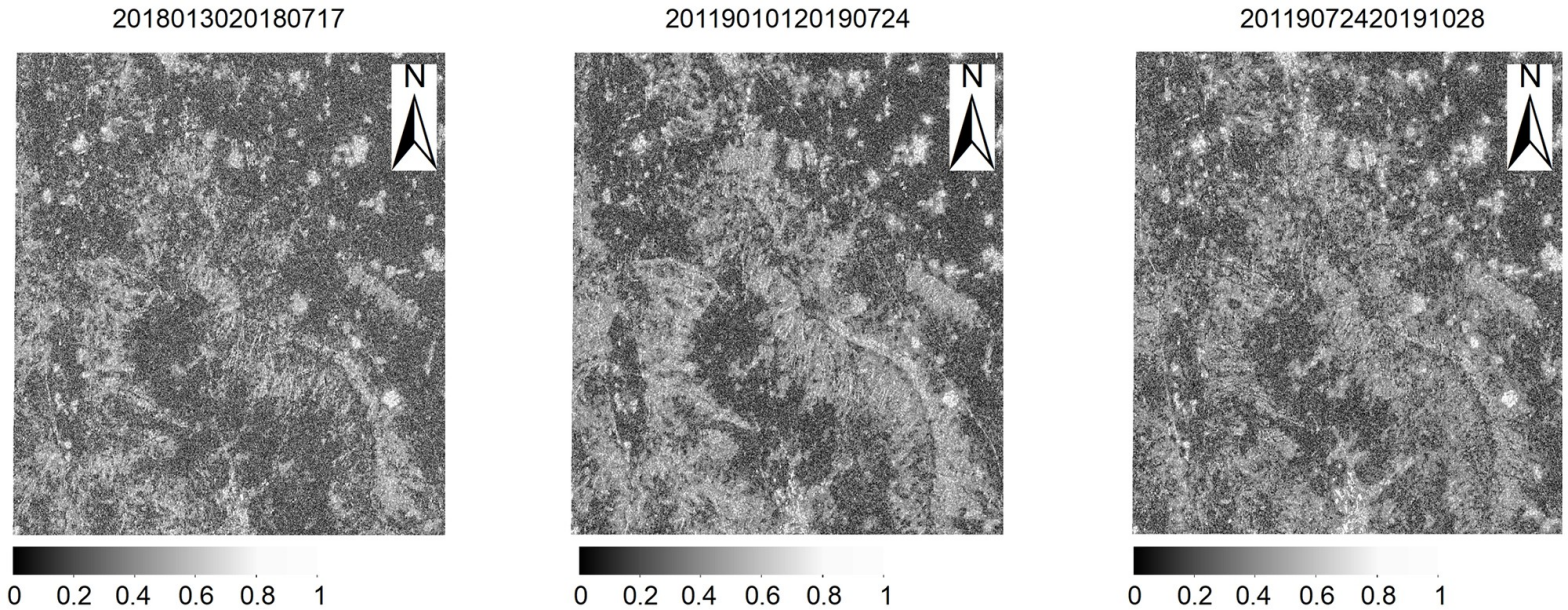
Part period coherence coefficient.

To optimize the interferometric combinations for a better quality of the interferometric phase, we calculate the interferometric coherence maps and the average interferometric coherence coefficients of 135 interference pairs with 30 Sentinel-1A images. Part of coherence coefficient was shown in the Fig 5.

## Results and analysis

### Analysis of subsiding rate

**Fig 6:**
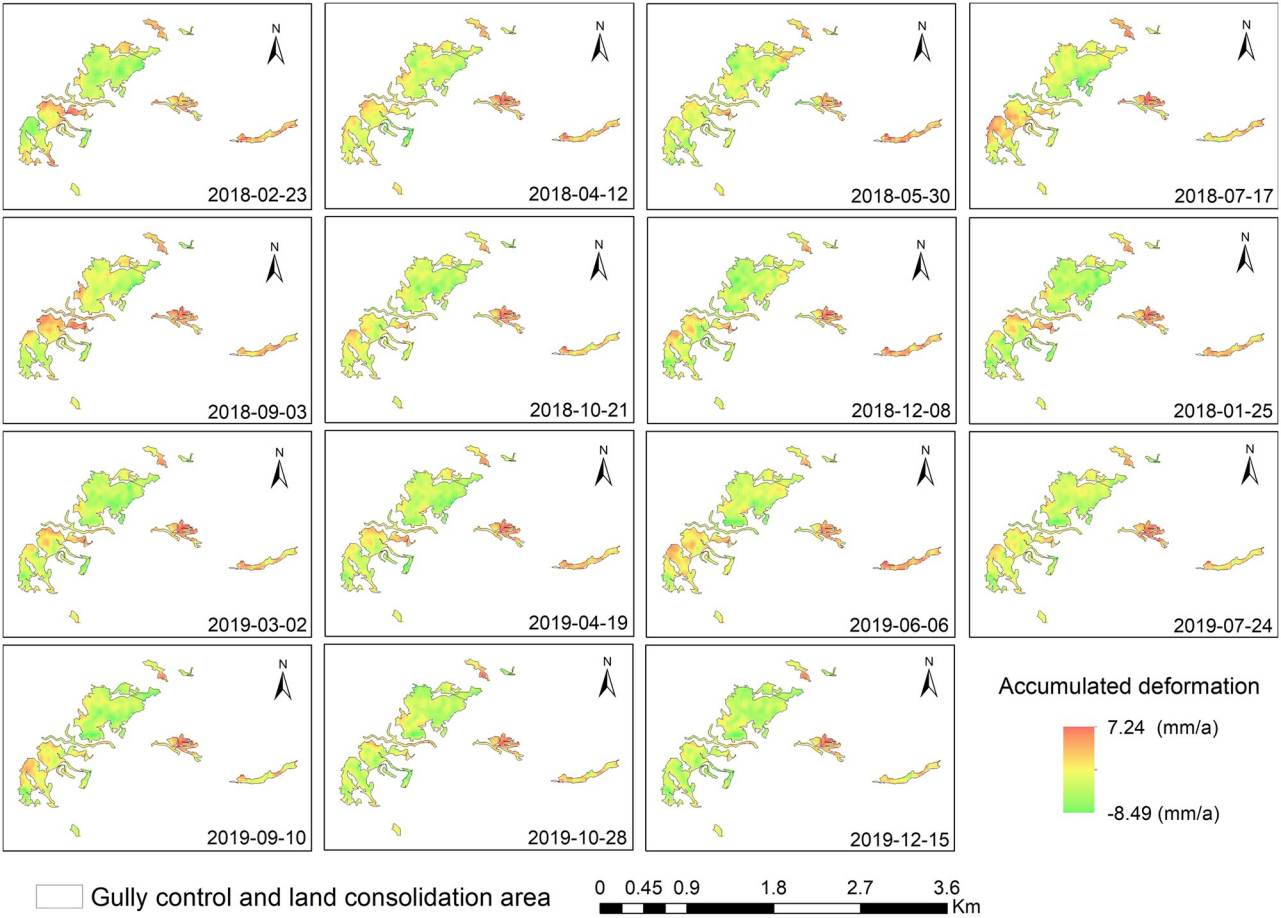
Time-series surface accumulated deformation in the target area in different periods.

In order to reveal the spatial and temporal evolution characteristics of the ground deformation in gully control and land consolidation in hilly loess region of the Loess Plateau from 2018 to 2020, time series graphs of accumulated deformation were drawn. (See Fig 6).

As can be observed from Fig 6, the accumulated deformation has increased with time and the influence scope continued to expand. Accumulated deformation rate was within -8.49 mm∙a^-1^ to 7.24 mm∙a^-1^. No significantly higher subsidence or uplift is generated in the area. From April to August, accumulated deformation of gully control and land consolidation area was low, but from September to March of the next year, accumulated deformation was obviously increased. That reflects that the infiltration of rainwater can lead to compacted loess deformation which caused by moistening effect. In terms of accumulated deformation spatial distribution, the accumulated deformation rate of the east part approach to maximum 7.24 mm∙a^-1^ higher than west part approach to minimum -8.49 mm∙a^-1^. Additionally, accumulated deformation rate at edge was larger than that at the central part in the land consolidation area.

**Fig 7:**
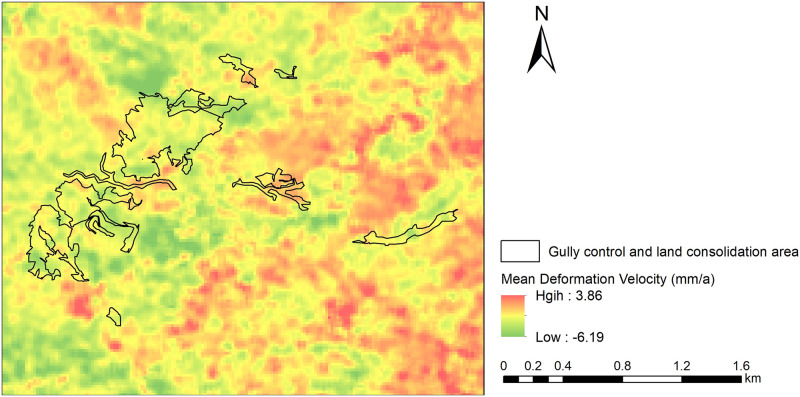
Mean Deformation Velocity of gully control and land consolidation area. Source: USGS Earth Resources Observatory and Science Center (https://www.usgs.gov/centers/eros).

The Mean Deformation Velocity of gully control and land consolidation area is shown in Fig 7.

In the Fig 7, red color represents the negative value of deformation velocity, indicating that land subsidence which the ground object moves away from the satellite along the direction of radar sight; green color represents the positive value of deformation velocity, indicating that land uplift which ground object moves close to the satellite along the direction of radar sight.

Land subsidence is the main deformation in land consolidation area, only a small part area is land uplift. The mean annual velocity of subsidence was from -6.19 mm∙a^-1^ to 3.86 mm∙a^-1^. The subsidence rate increased with the decreasing distance from the original gully center. The east part of the target area exhibited slight uplift: the maximum annual velocity of ground uplift was 3.86 mm∙*a*^-1^. Because of this area is excavation division, elastic strain energy of original loess under the excavation surface can rapid released which generated unloading rebound deformation after excavation of the upper mountain is the main internal mechanism of ground uplift in this area. The distribution of land subsidence in the land consolidation area is significantly related to fill thickness.

### Evaluation of land subsidence accuracy

**Fig 8:**
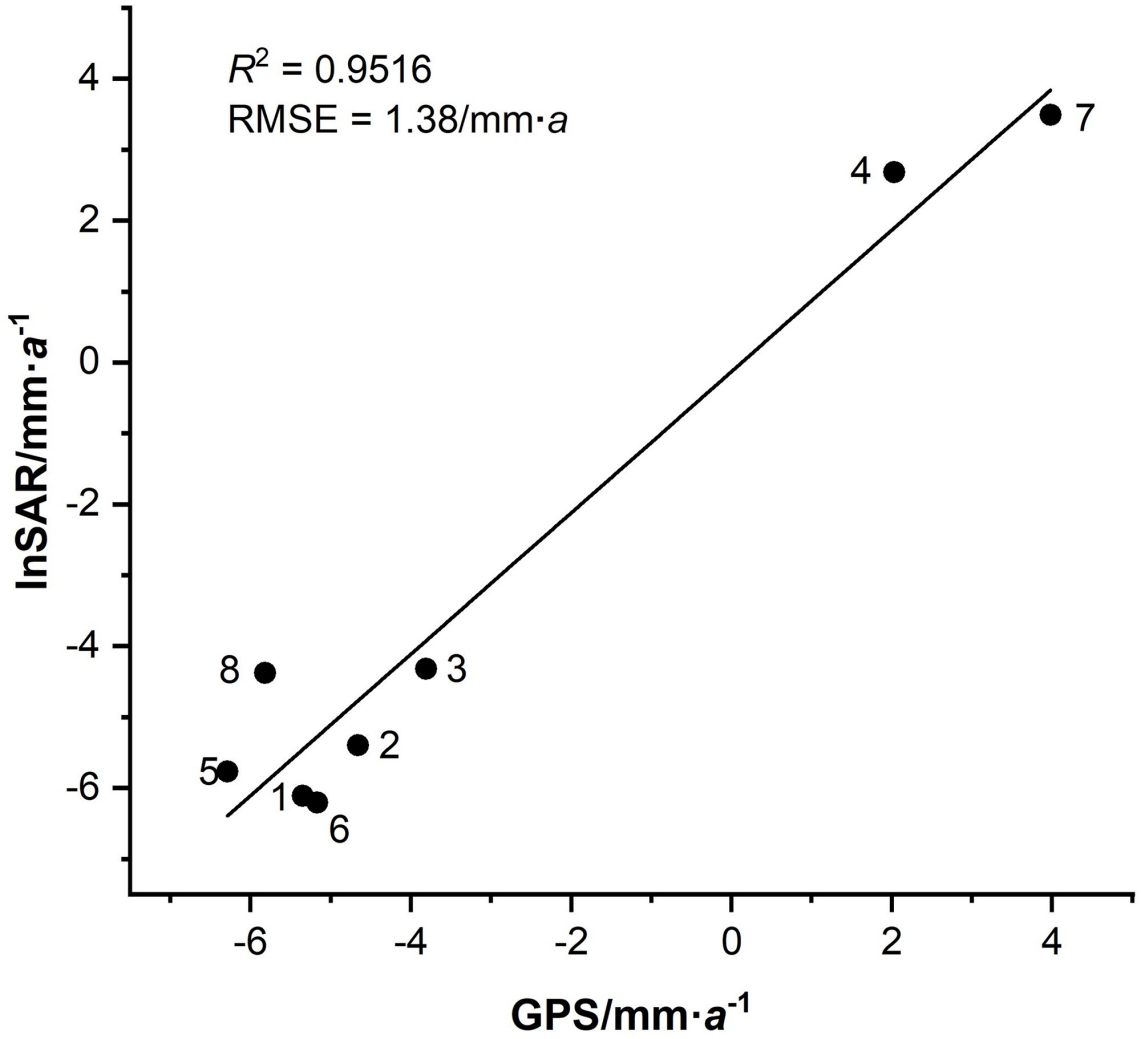
Comparison between time series of InSAR processing results and GPS measurements.

The results of evaluation of land subsidence accuracy are shown in Table 2 and Fig 8.

**Table 2:** Comparison between time series of InSAR processing and GPS measurements results.

GPS station	1	2	3	4	5	6	7	8
InSAR processing results /mm∙*a*^-1^	-6.11	-5.40	-4.32	2.68	-5.77	-6.21	3.49	-4.38
GPS observation results /mm∙*a*^-1^	-5.35	-4.66	-3.81	2.03	-6.29	-5.17	3.98	-5.82
Difference /mm∙*a*^-1^	0.76	0.74	0.51	-0.65	-0.52	1.04	0.49	-1.44

As can be observed from Table 2 and Fig 8, the maximum absolute error is 1.04 mm∙*a*^-1^, the minimum absolute error is -1.44 mm∙*a*^-1^. And the RMSE between the GPS measurements values of each station and the InSAR processing results is 1.38 mm∙*a*^-1^. Comparison indicating good consistency between the InSAR processing results and GPS measurements. The *R*^2^ between the GPS measurements values of each station and the InSAR processing results is 0.9516, which indicating that the accuracy of mean deformation velocity of sight direction obtained by InSAR is high.

### Effects of precipitation

**Fig 9:**
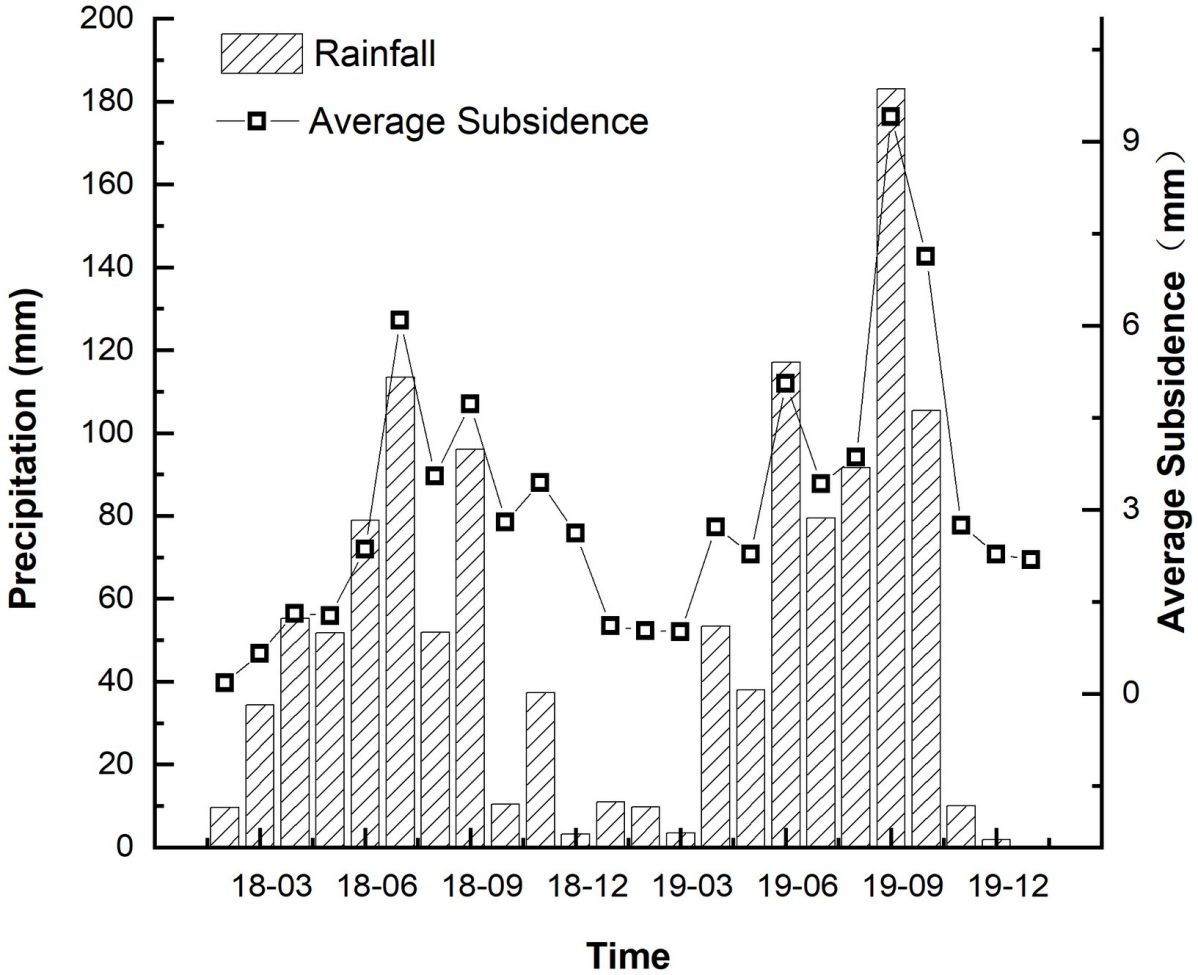
Subsidence in the target area vs. monthly erosive precipitation.

Precipitation is one of the main causes for soil erosion. If one-time precipitation exceeds 12.7 mm, soil erosion is 149 readily observed, and such precipitation is called erosive precipitation. To avoid the randomness of single points, 10 random points (close to each other) in the target area were selected, and their average subsidence was defined as the accumulated subsidence of each month. Based on precipitations in the target area at different intervals, the effects of monthly precipitation on point subsidence were investigated (Fig 9).

In hilly loess regions in the Loess Plateau, which are exposed to arid and semi-arid climates, moisture in soil evaporates continuously with no replenishment from precipitation, and capillary actions lead to moisture concentration near the contact points of coarse particles [23]. Additionally, fine particles (e.g., powders and clay particles) and some water-soluble salts gradually concentrated to the contact points of coarse particles, resulting in cemented sediment [24]. Affected by precipitation, soil layers 2~3 m from the surface has moderate humidity. During land leveling, soil layers 2~3 m from the surface in certain areas had practical compactness that was lower than that at under-consolidation state, resulting in severe gravity-compacted consolidation. Before completion of gravity-compacted consolidation, soil layers had loose structures. In case of large precipitation, surface water seeps into the ground, resulting in reduced compactness and increased humidity of the soil layer. Meanwhile, surface was exposed to collapse deformation. Upon the presence of external loads, soil layer structure was damaged and soil strength degraded drastically, resulting in land subsidence.

As shown in Fig 9, average time series of subsidence significantly accelerated with the increase of precipitation. Precipitation has certain effect which is temporary on surface subsidence in the research area. Subsidence gradually tended to the original normal consolidation subsidence after the rainy season finished. However, subsidence has significantly increased in the unexpected strong precipitation. This process accelerated the subsidence reflects that the infiltration of rainwater can lead to compacted loess deformation which caused by moistening effect.

### Effects of ground water

The ground water is the main source of irrigation water and one of the direct causes of Land subsidence in the target area. The ground water flow direction is from northeast to southwest. The total amount of water resources in the research area is 166 million m^3^, including surface water resources of 6 million m^3^, groundwater resources of 13 million m^3^. Thickness of aquifer is 10-120m, and depth of groundwater level is 50-120m. Water outflow from single well is generally 2–15 m^3^/ h. Many wells in the target area are major sources of irrigation water. From Dec to Aug, water was extracted from these wells for frequent and large-scale irrigation, while the consumption of irrigation water in winter was slightly higher than that in spring. The correlation between groundwater level and subsidence was analyzed by regression model. The correlation coefficient *R*^2^ of regression model was closer to 1 demonstrates that the correlation between groundwater level and land subsidence is with high correlativity. The correlation coefficient *R*^2^ of water intake point 1 and point 2 are 0.906 (P < 0.001) and 0.895 (P < 0.001). The two water intake points are closer to 1 demonstrates that subsidence is positively correlated with groundwater level.

**Fig 10:**
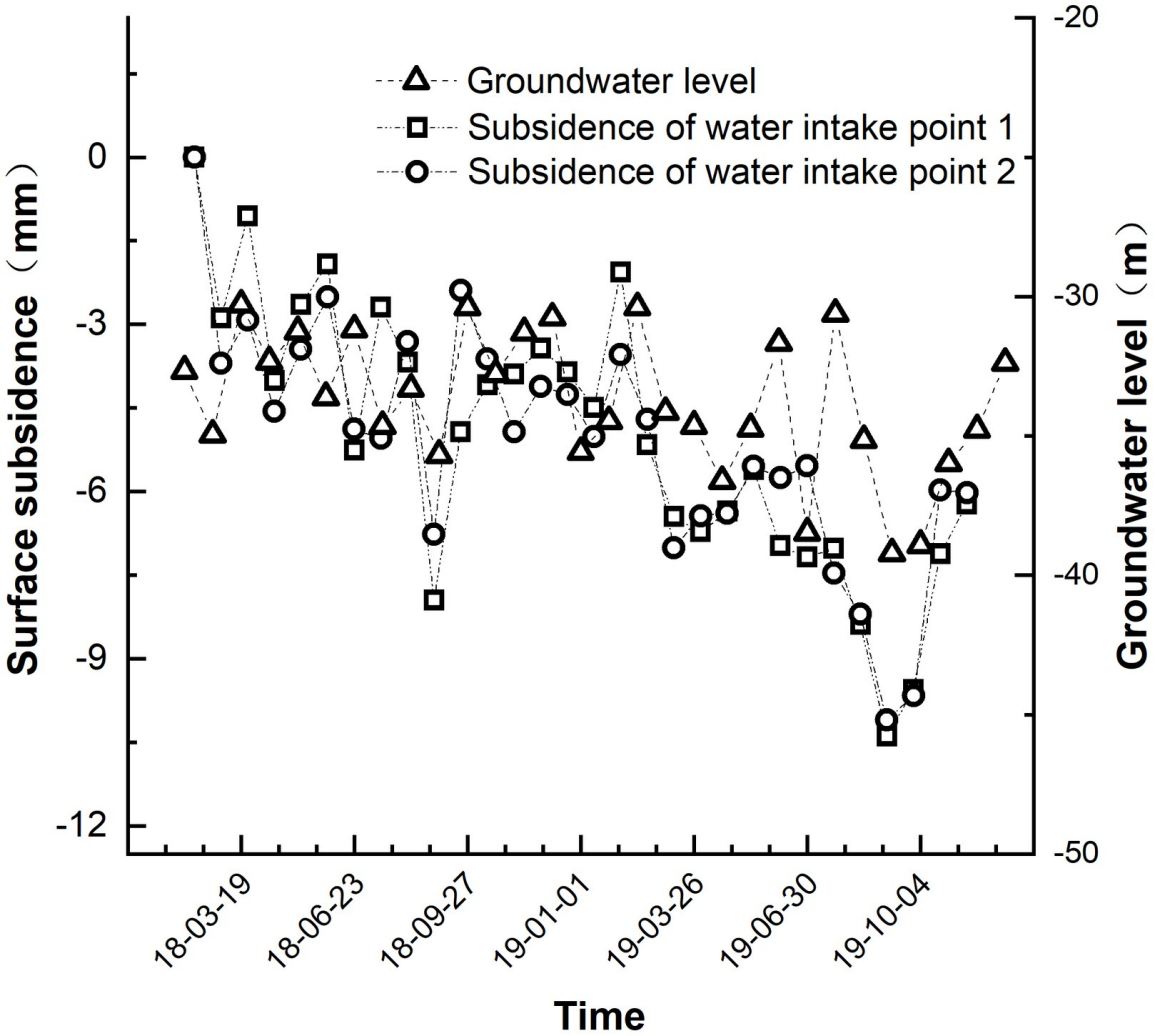
Water level at sampling point vs. ground water table.

As observed in Fig 10 the ground water table exhibited a trend like that of subsidence, demonstrating seasonal groundwater changes also cause seasonal surface uplift. Precipitation is the main source of groundwater recharge in the gully control and land consolidation in hilly loess regions, but the original terrain in the area is rugged, so precipitation is easily directly transformed into surface runoff, which reduces the ability to replenish groundwater. As a result, the groundwater level in the region is low and unevenly distributed. However, after the implementation of the gully control and reclamation project, the terrain became generally flat, and it was difficult for precipitation to be converted into surface runoff, resulting in a great increase in groundwater infiltration. Areas exposed to gully control and land consolidation in hilly loess regions in the Loess Plateau require a large amount of filling soil and exhibits complicated construction environment due to interactions of geological, hydrological, and climatic factors. Under varying ground water environment, external loads may lead to soil collapse, resulting in non-uniform Land subsidence.

### Effects of loess engineering properties

Original loess and compacted loess in the gully control and land consolidation region were taken samples. The physical properties at the different location’s samples were test in laboratory. The results are shown in Table 3.

**Table 3:** Main physical properties of original loess and compacted loess at different locations.

Sample point	Natural density *ρ g* · *cm*^−3^	Moisture *ω* %	Dry density *g* · *cm*^−3^	Specific gravity *G*_*s*_	Void-ratio *e*	Plastic limit *ω*_*p*_ %	Liquid limit *ω*_*L*_ %	Plasticity index *I*_*p*_ %
Original loess 1	1.76	9.85	1.59	2.68	0.65	15.85	31.61	15.88
Compacted loess 1	1.78	16.62	1.55	2.59	0.66	19.40	29.29	11.36
Compacted loess 2	1.82	12.60	1.60	2.66	0.63	17.50	30.08	12.51
Compacted loess 3	2.03	18.03	1.72	2.62	0.53	19.38	33.55	14.17

As observed in Table 3, physical properties of samples at different location are also different. It demonstrates physical properties have changed by remolded loess in gully control and land consolidation. The homogeneous physical properties were unable to perform in excavation and filling engineering. Therefore, the difference of physical properties of compacted loess leads to different subsidence.

**Fig 11:**
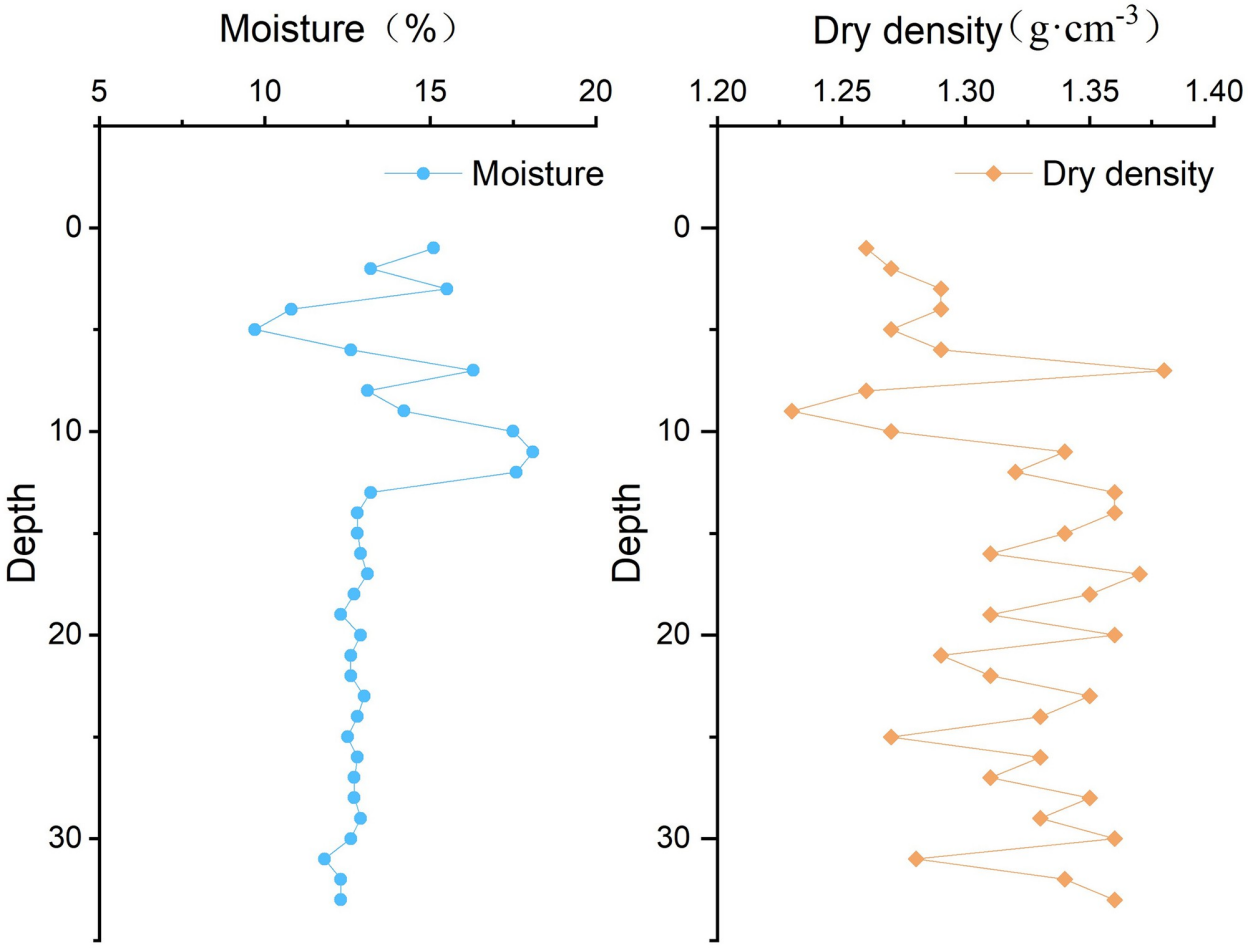
Comparison of moisture and dry density of compacted loess in different depth.

Comparison of moisture and dry density of compacted loess in different depth is shown in Fig 11.

As observed in Fig 11, moisture and dry density of compacted loess is discontinuity change with increasing depth that give rise to compacted loess of physical and mechanical properties is diversity in different depth. Accordingly, compacted loess compression deformation also diversity in different depth. Eventually, inhomogeneous subsidence generated in the gully control and land consolidation.

## Discussion

The slope soil in areas exposed to gully control and land consolidation in hilly loess region of the Loess Plateau is dominated by backfill compacted loess induced by short-term restructuring and its performance was affected by soil texture, water content, dry density, consolidation time, and overburden pressure. As an artificially compacted loess, the slope loess in the target area was textured and contained a considerable number of fine particles or gravels. Owing to varying bottom layer induced by geological agent, this compacted loess tended to form soil layers with non-uniform thickness distribution and significantly varying consolidation under the sedimentation effect at the initial stage, consolidation. The filling soil in the target area exhibited poor diagenesis, resulting in high compressibility [25].

The natural development of loess strata is a long process, including compression, collapsible deformation and consolidation compaction. The practice of soil mechanics suggested that loess compressibility can be mainly attributed to water-induced collapse and dehydration consolidation [26]. In the presence of large precipitation, water infiltration led to partial loss of fine particles in the loess layer. Under pressures, loess may be exposed to structural damage in presence of water, resulting in additional subsidence. This phenomenon is called collapse. After the rainy season, ground water loss accompanied by moisture evaporation was observed, resulting in significant changes of soil permeability. Under soil gravity and external loads, tensile stress of the soil increased, resulting in dry shrinkage of soil and micro-cracks in the soil layer. This phenomenon is regarded as dehydration consolidation [27].

Compacted loess is stratification soil contains more fines or gravel. Because it was reconstructed in short term, its property was influenced by a lot of factors such as soil texture, moisture content, dry density, consolidation time, overlying soil pressure. Moreover, the compacted loess in land consolidation area have only developed for three years (2015–2017), formation time of the compacted loess structure was short, the stability of loess structure was poor, compression deformation is not yet completely stable, the soil particles are loosely arranged, and the bearing capacity of external load is weak. So, the collapsibility of compacted loess is more than original loess. In the gully control and land consolidation region, the original loess layer covers a compacted loess layer with the thickness of about 100 m. Accordingly, consolidation subsidence more likely to occur in land consolidation region were influenced of external factors.

**Fig 12:**
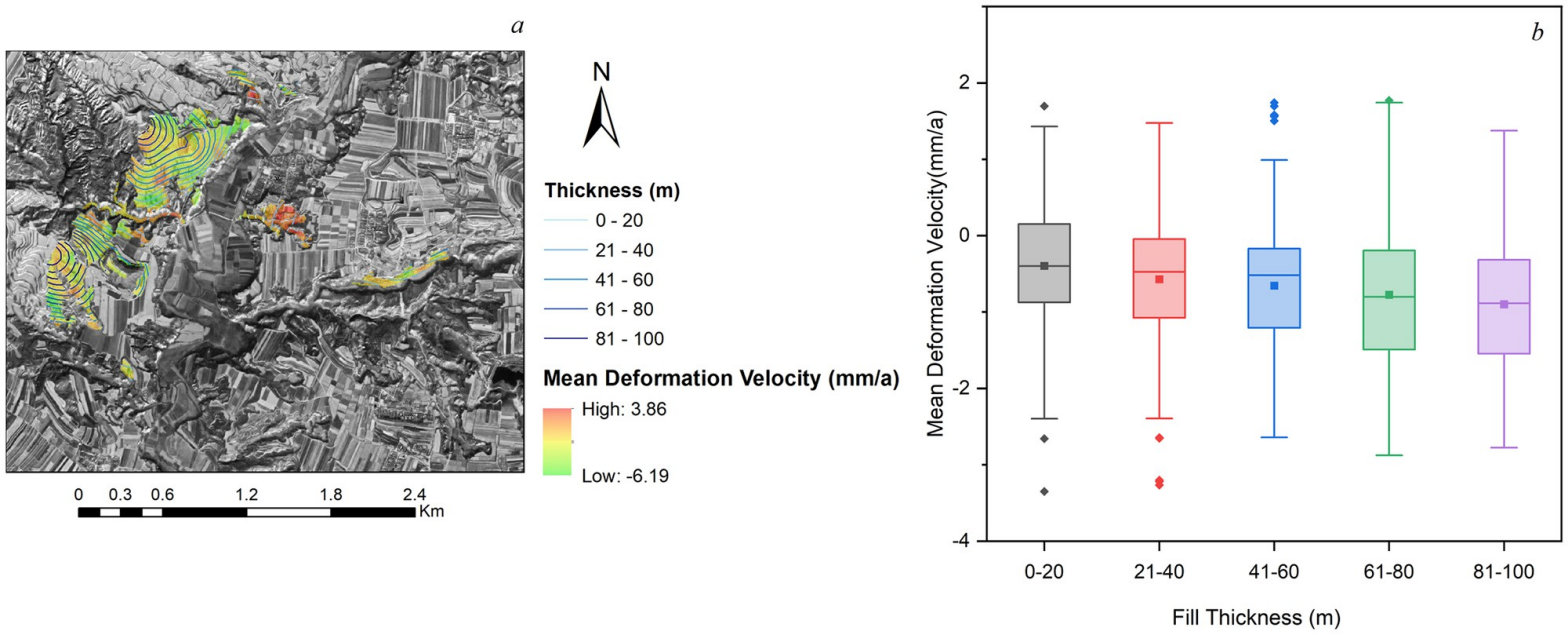
Subsidence rate and fill thickness in gully control and land consolidation.

Large amount of compacted loess was generated by excavation and backfill ditch. The result of comparison of subsidence velocity and fill thickness was shown in the Fig 12a. In order to further analyze the relationship between subsidence rate and fill thickness, statistical analysis for fill thickness of all deformation velocity points in the land consolidation region was shown in the Fig 12b.

As shown in the Fig 12a, fill thickness of high subsidence is greater than 60 m demonstrate that distribution and thickness of fill have significantly effects on subsidence distribution and rate of gully control and land consolidation. Fig 12b shows the subsidence velocity under different filling thickness. The square in the middle of the box plot represents the average number of data which can represent average level of subsidence rate in consolidation area. As shown in Fig 12b, mean subsidence velocity of five filling thickness (0–20 m, 21–40 m, 41–60 m, 61–80 m, 81–100 m) approximately is -0.43 mm∙a^-1^, -0.58 mm∙a^-1^, -0.69 mm∙a^-1^, -0.77 mm∙a^-1^, -0.82 mm∙a^-1^. The mean subsidence velocity increased obviously with the increase of filling thickness. Filling thickness controls the magnitude of subsidence rate. The greater the filling thickness, the higher the subsidence rate. It is known in analysis for effects of loess engineering properties, Physico-mechanical property of compacted loess is different in various depths. So, self-weight compression deformation of compacted loess in various depths lead to underlying original loess pressure varied with fill thickness. The greater the fill thickness, the greater the fill self-weight compression deformation, and the greater underlying original loess pressure. In general, according to the comprehensive comparative analysis of land subsidence rate and fill thickness in the research area, it is reasonable to conclude that co-compression deformation of original loess and compacted loess is main influencing factors of subsidence.

Different incidence angles which caused by ascending orbits descending orbits induce SBAS-InSAR LOS direction are also different. Furthermore, there is still a limitation in ground deformation monitoring by SBAS-InSAR. The true horizontal direction displacements of the ground have been improperly converted to the vertical direction that increased the error of vertical deformation observation. The ground deformation mainly in gully control and land consolidation in hilly loess region of the Loess Plateau is in the vertical direction, while ground deformation in horizontal direction is very small. Therefore, ground deformation in horizontal direction can be ignored. The question of whether the deformation in the LOS direction reflects the true displacement of the ground has been addressed in many research [28–30]. Due to the monitoring results are obtained based on a single data source and a single InSAR processing method, it is difficult to obtain the real 3-D ground deformation. However, combining the available relevant theories and the geometric relationship between SAR satellites and monitoring sites (mainly concerning slope and aspect), we found that the deformation in LOS direction is consistent with the true ground displacement. InSAR processing results and GPS measurements was high consistency also supports the reliability of the results. The correlation between slope and deformation velocity needs to be investigated in depth in the follow-up to propose slope threshold of slope when the anomalies occurred, which will allow us to better analyze the true ground displacements.

SBAS-InSAR can be applied to monitoring the precursor information of mountain or underground geological hazards [31]. The potential of the SBAS-InSAR can be highlighted from these broad spectra of SBAS applications, such as ground subsidence, earthquake, landslides, permafrost degradation, and glacier movement. However, the SBAS-InSAR also faces a series of problems, such as deformation measurement in low-coherence regions, estimation of atmospheric errors, and data processing in the big data era. In the big data era, the SBAS-InSAR will be developed for high-precision, large-area, long-time sequences, and real-time surface deformation monitoring.

## Conclusions

Based on the 30-scene Sentinel-1A IW SLC data, land subsidence monitoring of areas exposed to gully control and land consolidation in hilly loess region of the Loess Plateau in 2018–2019 was achieved by the SBAS-InSAR method. The annual rate of land subsidence and accumulated subsidence in the target area were obtained and the spatial-temporal distribution of land subsidence in the target area and its subsiding mechanism were analyzed. The following conclusions can be drawn:

The gully control and land consolidation in hilly loess area of the Loess Plateau has generated inhomogeneous subsidence but there is no significant subsidence or uplift in the area. The accuracy of mean deformation velocity of sight direction obtained by InSAR is high. Compacted loess is a kind of reconstructed soil, difference in physical and mechanical properties is main internal factor of subsidence. The main control factor of subsidence of distribution and size is fill thickness. SBAS-InSAR can provide effective technology for early identification of potential hazards and active risk prevention in hilly loess area of the Loess Plateau and provide evidence for subsidence and side slope slip monitoring and warning.
